# An Unusual Case of a Stuck Catheter in a Sick Neonate

**DOI:** 10.7759/cureus.71773

**Published:** 2024-10-18

**Authors:** Abhinav Aggarwal, Roshan Kumar, Alpas Anand, Preeti Gupta

**Affiliations:** 1 Internal Medicine, Guru Teg Bahadur Hospital, New Delhi, IND; 2 Interventional Cardiology, Vardhman Mahavir Medical College and Safdarjung Hospital, New Delhi, IND; 3 Cardiology, Vardhman Mahavir Medical College and Safdarjung Hospital, New Delhi, IND; 4 Pediatrics, Vardhman Mahavir Medical College and Safdarjung Hospital, New Delhi, IND

**Keywords:** broken catheter, cardiac catheterization, snare, stuck catheter, umbilical vein catheter

## Abstract

The umbilical vein can be reliably used for central venous access in neonates less than 14 days old. The catheter used in the umbilical vein normally extends proximally to the inferior vena cava and can be utilized for drug delivery as well as venous sampling. Herein, we describe a neonate with a broken umbilical vein catheter (UVC) stuck in the right atrium and inferior vena cava (IVC), and its successful, uneventful removal via the transfemoral route.

## Introduction

The umbilical vein can be used as a reliable venous access in sick neonates. Umbilical vein catheterization is a relatively safe procedure, although complications such as catheter infection or thrombosis may sometimes occur. However, rarely, the catheter may break during removal and become retained in the inferior vena cava and right atrium, leading to thrombus formation or embolization. The exact incidence of broken umbilical vein catheters is unknown, and there are only isolated case reports in the literature. Most cases have been removed by surgical exploration or transcatheter removal using a snare [1]. A stuck catheter is an emergency, and transcatheter removal using a snare via femoral venous access is an innovative approach to address this rare complication.

## Case presentation

**Figure 1:**
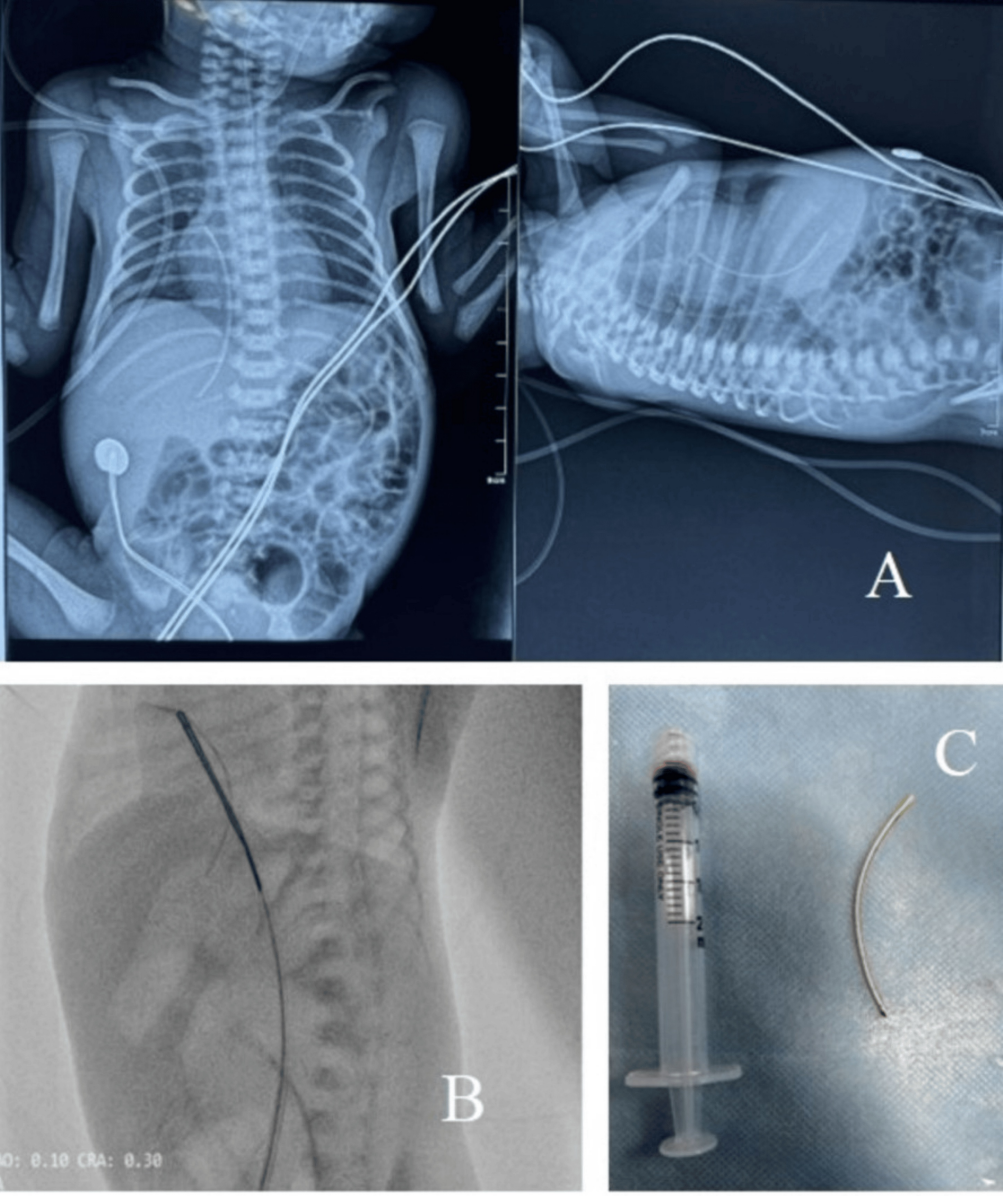
(A) Anteroposterior (AP) and lateral chest radiograph showing the stuck umbilical vein catheter in the right atrium (RA) and inferior vena cava (IVC). (B) Fluoroscopy showing the catheter being snared out using femoral access. (C) Removed broken fragment of the umbilical vein catheter.

A preterm male neonate weighing 2 kg was referred to the cardiology department of our hospital with a stuck umbilical vein catheter. A 3.5 Fr umbilical vein catheter (UVC) was inserted on day 1 of life for drug delivery at an outside hospital where the neonate was born. On the 10th day of his life, after his general condition improved, removal of the catheter was attempted. However, during the removal, the UVC broke, and all attempts to retrieve the broken portion of the UVC through local exploration failed. A radiograph showed the broken umbilical vein catheter in the heart, in the inferior vena cava (IVC), and in the right atrium (Figure 1A).

We planned to attempt a transcatheter removal, as it is less invasive and avoids the risks associated with surgical exploration. Under local anesthesia, trans-femoral venous access was obtained using a 6 Fr sheath. A 20 mm snare was used through a 6 Fr catheter to grasp the broken catheter in the right atrium and retrieve it successfully (Figure 1B). The patient did not experience any procedural complications. Once venous access was obtained, the procedure took 10 minutes to snare out the broken fragment. Later, the neonate was discharged the next day, as there were no procedural complications. The child is doing well on follow-up.

## Discussion

A UVC is a small, flexible tube inserted into the umbilical vein through the umbilical stump in newborn babies. It is a standard procedure in neonatal intensive care units for delivering medications, fluids, and blood sampling. The most common complication of UVC placement is infection, with the risk increasing when the catheter is left in place for extended periods. Once the catheter is no longer needed, it is removed by gently pulling it out. Care must be taken to ensure the catheter is intact and that undue force is not applied during removal. Rarely, the catheter may break while being pulled out [1].

The main risks associated with transcatheter retrieval are related to venous access and the possibility of dislodging the fragment further if snaring is not performed correctly. These risks can be mitigated by using a smaller sheath size and employing correct snaring techniques. The stuck catheter is a rare complication that can be successfully managed via a transcatheter route using a snare to retrieve the broken fragment if local exploration is unsuccessful [2].

## Conclusions

A stuck umbilical vein catheter is a rare complication that can be successfully managed via a transcatheter route using a snare to retrieve the broken fragment if local exploration is unsuccessful.

## References

[1] Khasawneh W, Samara DN, Bataineh ZA (2021). Umbilical catheter rupture: a serious complication in neonatal intensive care units. Int J Pediatr Adolesc Med.

[2] Dhua AK, Singh B, Kumar D, Awasthy N (2013). Broken umbilical vein catheter as an embolus in a neonate - an unusual preventable complication. J Neonatal Surg.

